# Chronic Residential Exposure to Particulate Matter Air Pollution and Systemic Inflammatory Markers

**DOI:** 10.1289/ehp.0800362

**Published:** 2009-05-11

**Authors:** Barbara Hoffmann, Susanne Moebus, Nico Dragano, Andreas Stang, Stefan Möhlenkamp, Axel Schmermund, Michael Memmesheimer, Martina Bröcker-Preuss, Klaus Mann, Raimund Erbel, Karl-Heinz Jöckel

**Affiliations:** 1 Institute for Medical Informatics, Biometry, and Epidemiology, University of Duisburg-Essen, Essen, Germany; 2 Institute of Medical Sociology, University of Düsseldorf, Düsseldorf, Germany; 3 Institute of Medical Epidemiology, Biometry, and Informatics, Martin-Luther-University of Halle-Wittenberg, Halle, Germany; 4 West German Heart Center Essen, University of Duisburg-Essen, Essen, Germany; 5 Rhenish Institute for Environmental Research at the University of Cologne, Cologne, Germany; 6 Department of Endocrinology and Division of Laboratory Research, University of Duisburg-Essen, Essen, Germany

**Keywords:** air quality, cardiovascular disease, epidemiology, inflammation, roadway proximity

## Abstract

**Background:**

Long-term exposure to urban air pollution may accelerate atherogenesis, but mechanisms are still unclear. The induction of a low-grade systemic inflammatory state is a plausible mechanistic pathway. Objectives: We analyzed the association of residential long-term exposure to particulate matter (PM) and high traffic with systemic inflammatory markers.

**Methods:**

We used baseline data from the German Heinz Nixdorf Recall Study, a population-based, prospective cohort study of 4,814 participants that started in 2000. Fine PM [aerodynamic diameter ≤ 2.5 μm (PM_2.5_)] exposure based on a small-scale dispersion and chemistry transport model was assigned to each home address. We calculated distances between residences and major roads. Long-term exposure to air pollution (annual PM_2.5_ and distance to high traffic) and concentration of inflammatory markers [high-sensitivity C-reactive protein (hs-CRP) and fibrinogen] on the day of the baseline visit were analyzed with sex-stratified multiple linear regression, controlling for individual-level risk factors.

**Results:**

In the adjusted analysis, a cross-sectional exposure difference of 3.91 μg/m^3^ in PM_2.5_ (interdecile range) was associated with increases in hs-CRP of 23.9% [95% confidence interval (CI), 4.1 to 47.4%] and fibrinogen of 3.9% (95% CI, 0.3 to 7.7%) in men, whereas we found no association in women. Chronic traffic exposure was not associated with inflammatory markers. Short-term exposures to air pollutants and temperature did not influence the results markedly.

**Conclusions:**

Our study indicates that long-term residential exposure to high levels of PM_2.5_ is associated with systemic inflammatory markers in men. This might provide a link between air pollution and coronary atherosclerosis.

Chronic elevated levels of particulate matter (PM) air pollution and long-term residential exposure to high traffic levels both increase cardiovascular morbidity and mortality (Beelen et al. 2008; Brook et al. 2004; Miller et al. 2007; Rosenlund et al. 2006). In animal experiments, long-term exposure to high PM induces the development and progression of atherosclerosis, the major underlying pathology for cardiovascular disease (Sun et al. 2005), with stronger effects seen for traffic-related ultrafine particles (UFPs) (Araujo et al. 2008). Initial epidemiologic evidence indicates an association between air pollution and traffic and atherosclerosis in humans as well (Diez Roux et al. 2008; Hoffmann et al. 2007; Künzli et al. 2005).

Uncertainty exists about the mechanisms involved in the association between long-term exposure to PM and atherosclerosis. Development and progression of atherosclerosis are strongly linked to inflammatory mechanisms (Libby 2002). Plausible mechanisms for the effect of air pollution on atherogenesis include the induction of low-grade pulmonary inflammation with a secondary systemic inflammatory response. Alveolar macrophages and bronchial epithelial cells exposed to PM release increased quantities of cytokines and chemokines (van Eeden et al. 2001), which enter the systemic circulation and stimulate the production of acute-phase proteins such as C-reactive protein (CRP) and fibrinogen in the liver. High levels of high-sensitivity (hs)-CRP and fibrinogen are independent predictors of cardiovascular disease (Fibrinogen Studies Collaboration 2007; Ridker and Morrow 2003) and may also act as mediators in the pathogenesis of atherosclerosis due to air pollution (Suwa et al. 2002). It has been shown that increased levels of CRP resulting from exposure to PM are involved in the infiltration of monocytes into the arterial wall (Yatera et al. 2008), which promotes atherogenesis by amplifying inflammatory and pro-coagulant responses (Libby 2002).

There is only limited epidemiologic evidence for the association between PM and systemic inflammatory markers in the general population. Some investigators have observed associations between short-term increases in PM and CRP or fibrinogen (Pekkanen et al. 2000; Schwartz 2001), whereas others have found no consistent or only weak associations (Baccarelli et al. 2007a; Diez Roux et al. 2006; Liao et al. 2005; Steinvil et al. 2008) or associations limited to selected populations (Dubowsky et al. 2006; Peters et al. 2001; Rückerl et al. 2007; Seaton et al. 1999; Yue et al. 2007). A few studies so far have shown evidence for associations between medium- or long-term PM exposure (weeks to months) and inflammatory markers suggesting a sustained inflammatory response due to continuously or repeatedly elevated PM (Chen and Schwartz 2008; Diez Roux et al. 2006; Zeka et al. 2006), whereas others did not find an association (Forbes et al. 2009).

In this study, we examined whether long-term residential exposure to high levels of urban air pollution is associated with hs-CRP and fibrinogen, two systemic inflammatory markers, independent of short-term changes in air pollution and meteorology. We make use of a well-examined population-based cohort located in a highly industrialized area of Western Germany.

## Materials and Methods

### Study design

We used baseline data from the Heinz Nixdorf Recall Study, an ongoing population-based, prospective cardiovascular cohort study that started in 2000 and includes 4,814 randomly selected participants 45–75 years of age from three large adjacent German cities (Essen, Mülheim, and Bochum) of the densely populated and highly industrialized Ruhr area. The study design has been described in detail elsewhere (Schmermund et al. 2002). It was approved by the relevant institutional ethics committees and follows strict internal and external quality assurance protocols. All study participants gave informed consent before the examinations. The baseline assessment included a self-administered questionnaire, face-to-face interviews for personal risk factor assessment, blood pressure measurements, anthropometric measurements, and comprehensive clinical and laboratory tests according to standard protocols.

### Exposure assessment

The study area covers a region of approximately 600 km^2^. We used the EURAD (European Air Pollution Dispersion) model, a dispersion and chemistry transport model (Memmesheimer et al. 2004) to estimate the annual mean values for background PM_2.5_ (aerodynamic diameter ≤ 2.5 μm) concentrations (year 2002) on a spatial scale of 5-km grid-cell length and assigned these to the addresses of the participants (ArcGIS software; ESRI, Redlands, CA, USA). The EURAD model uses input data from official emission inventories on a scale of 1 km^2^, including industrial sources, household heating, traffic, and agriculture data on hourly meteorology and regional topography. Surface concentrations are calculated by dispersing emissions in horizontal strata, taking chemical reactivity and transport processes into account. From the model output, we calculated daily surface concentrations of air pollutants for a grid-cell length of 5 km and validated these by comparing the model-derived values with measured air pollution data from monitoring sites (correlation between modeled daily averages of PM_2.5_ and measured PM_2.5_ was 0.86–0.88, depending on season) (Memmesheimer et al. 2004).

Daily means for PM and ozone from one central monitoring station representing the citywide background concentration of pollutants were received from the North Rhine-Westphalia State Agency for Nature, Environment and Consumer Protection [Landesamt für Natur, Umwelt und Verbrasucherschutz (LANUV) NRW, Essen, Germany]. No routine PM_2.5_ monitoring had been conducted in the Ruhr area during the study period, so we used PM_10_ concentrations to assess daily exposures to PM. For 2000 through 2002, only data for total suspended particle (TSP) mass measurements were available, and were converted to PM_10_ by multiplying by the factor of 0.85 for 2000 and by 0.7 for 2001 and 2002, according to standard procedures (LANUV NRW). We set values less than the limit of detection (LOD; TSP, 10 μg/m^3^; ozone, 4 μg/m^3^) to LOD/2. Hourly temperature variables were obtained from the German National Meteorological Service (Deutscher Wetterdienst). For each individual blood draw, we assessed the daily means of PM_10_, ozone, and air temperature for the 5 days before the day of sampling.

We estimated residential exposure to traffic by the distances between residence and major roads (freeways and federal highways), using administrative digitized maps based on compulsory surveying and mapping of all buildings with a precision of at least 0.5 m (MapInfo GmbH, Raunheim, Germany).

### Markers of inflammation

As markers of inflammation, we measured high-sensitivity (hs)-CRP using an automated nephelometer (BN-II; Dade-Behring Inc., Deerfield, IL, USA) and fibrinogen in plasma with an automated BCS analyzer (Dade-Behring Inc.). All analyses were performed in the central laboratory of the University Hospital of Essen.

### Socioeconomic status and lifestyle-related covariates

Socioeconomic status (SES) was assessed based on educational attainment as recommended by the German Epidemiological Association (Ahrens et al. 1998). We classified education according to the International Standard Classification of Education as total years of formal education, combining primary schooling and vocational or professional education in one variable (United Nations Educational, Scientific, and Cultural Organization 1997). Four categories were defined with the highest category of ≥ 18 years of education (equivalent to a university degree) and the lowest category of ≤ 10 years (equivalent to a basic school degree and no vocational training). Economic activity was included as employed, retired, unemployed, and economically inactive (mostly homemakers). In a subgroup with information on income, we calculated equivalent monthly household income by dividing total net household income by weighted number of household members.

Smoking status was categorized as current daily smoker, current occasional smoker, cessation of smoking within last year, cessation of smoking more than one year ago, and never-smoker. For current smokers, we assessed the number of smoked cigarettes per day. For current and ex-smokers, we also assessed the lifetime cumulative exposure in pack-years. We combined environmental tobacco smoke (ETS) at home, at the workplace, or in other places in one variable. Anthropometric measurements (height, weight, waist circumference) were conducted according to standardized protocols. Physical activity was assessed by converting daily activities and regular exercise into metabolic equivalents. Regular alcohol intake was defined as any alcohol consumption at least 4–6 days per week.

We assessed personal characteristics that might have an effect-modifying role. Diabetes mellitus was defined as prior physician diagnosis of diabetes, taking an antidiabetic drug, having a blood glucose ≥ 200 mg/dL, or having a fasting blood glucose ≥ 126 mg/dL. Coronary heart disease (CHD) was defined as a self-reported history of a myocardial infarction or coronary intervention. We defined current medication with cardiovascular or antiinflammatory drugs that could influence the concentration of inflammatory markers as any medication that includes statins, nonsteroidal antiinflammatory drugs (NSAIDs), angiotensin-converting enzyme inhibitors (ACE inhibitors), or beta-blockers (Casiglia and Tikhonoff 2007).

### Statistical analysis

The entire study population consists of 4,814 participants. We performed analyses on a subgroup of the study population for whom measurements of inflammatory markers and complete information on covariates were available (*n* = 4,036). Participants with acute infections or acute exacerbations of inflammatory diseases (hs-CRP > 100 mg/L) were excluded from the analysis (*n* = 4), leaving 4,032 participants for the final analyses.

We used the Spearman correlation coefficient to examine the correlation between long-term PM_2.5_ exposure and residential proximity to traffic and between daily apparent temperature and daily air pollutant concentrations.

To assess the association between long-term measures of air pollution and inflammatory markers, we performed multiple linear regression analyses with the natural logarithm of hs-CRP and fibrinogen as the dependent variables. We entered the annual mean PM_2.5_ concentration on a continuous scale and categorized distance to roads in four classes: ≤ 50 m, 51–100 m, 101–200, and > 200 m. PM_2.5_ effect estimates are given for the concentration difference between the 10th and the 90th percentile of the PM_2.5_ distribution (3.91 μg/m^3^). To investigate the linearity assumption of the relation and examine the exposure–response relationship, PM_2.5_ was classified according to quartiles of exposure (Q1, 21.54 μg/m^3^; Q2, 22.59 μg/m^3^; Q3, 23.75 μg/m^3^) in separate analyses.

Because daily variations in PM concentrations have been shown to exert a short-term effect on blood markers of inflammation (Seaton et al. 1999), we included the centrally measured PM_10_ concentration in a separate analysis. We averaged PM_10_ over the 5 days preceding the blood draw to account for the time needed to induce a systemic response with hepatic production of inflammatory markers and because prior studies have shown stronger associations for longer averaging times (Dubowsky et al. 2006; Yue et al. 2007; Zeka et al. 2006). Ozone concentration and mean air temperature were also entered, because they have been shown to influence inflammatory markers as well (Liao et al. 2005; Schneider et al. 2008). We determined average times (2-day for ozone and 5-day for temperature) based on model fit (*R*^2^). In models with short-term exposures, we also added a variable for the long-term time trend and conducted analyses stratified by season. Because of missing data on daily pollutant concentrations, analyses including daily pollutants were restricted to a subgroup of 1,752 men and 1,716 women. To ensure comparability of effect estimates, all effects are expressed per interdecile range of the exposure metric.

**Figure 1:**
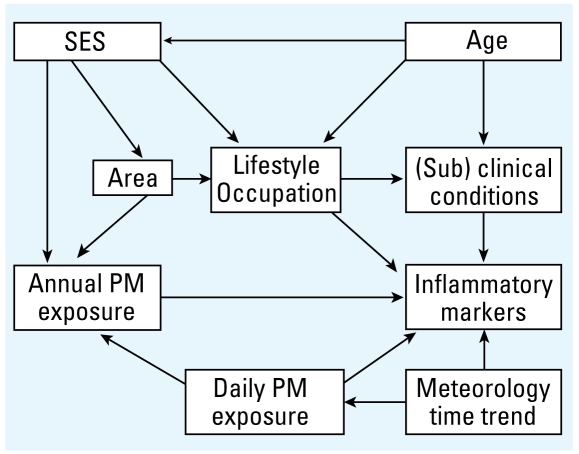
Causal diagram for the investigation of the relationship of residential air pollution exposure with inflammatory markers.

Although we based our analyses on cross-sectional data that do not contain information about the temporal relations between studied variables, we used causal diagrams [directed acyclic graphs (DAGs)] to identify the minimal sufficient adjustment set. Based on prior biological and epidemiological knowledge, we specified the most likely temporal relations between variables (Figure 1). According to this DAG, short-term PM exposure, SES, and area of residence need to be adjusted for (model 1). Because of the difficulties inherent in adjusting for such a broad construct as SES, which increases the possibility of residual confounding, we additionally identified a sufficient adjustment set (model 2) that does not include SES but includes age, area of residence, and lifestyle-related factors [smoking behavior, ETS, body mass index (BMI), waist circumference, physical activity, alcohol consumption, low-density lipoprotein (LDL), and high-density lipoprotein (HDL)]. We performed analyses using both adjustment sets.

Based on prior results, we used subgroup analyses to assess effect measure modification by age (< 60, ≥ 60 years), smoking status (regular current smokers, no regular current smoking), BMI (< 30 kg/m^2^, ≥ 30 kg/m^2^), diabetes, CHD, and intake of cardiovascular or antiinflammatory medication.

### Sensitivity analyses

We investigated the sensitivity of our results to alternative ways of modeling by (*a*) performing a multiple logistic regression analysis with hs-CRP and fibrinogen dichotomized according to the 90th percentile of their respective distributions (6.8 mg/L for hs-CRP and 438 mg/dL for fibrinogen); (*b*) using different lags and averaging times for recent exposure to PM_10_, ozone, and temperature (lags 1–5, averaging times to include the prior 2–5 days); and (*c*) analyzing whether the observed associations were stronger in participants who had not been working full time during the last year before the baseline analysis and therefore presumably had spent more time at home. We also explored the possible effects of medication use and comorbidities as well as the sensitivity of the results to the exclusion of area and city of residence as confounders, because adjusting for area might remove some of the effect of the spatially distributed long-term exposure.

## Results

**Figure 2:**
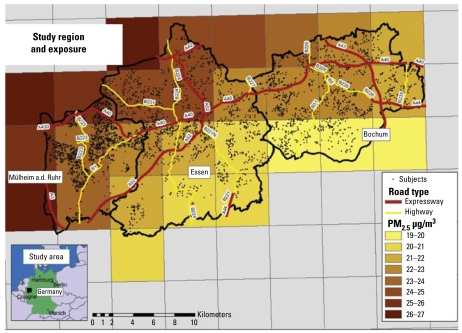
Study region of the Heinz Nixdorf Recall Study: annual PM_2.5_ values for the year 2002.

**Figure 3:**
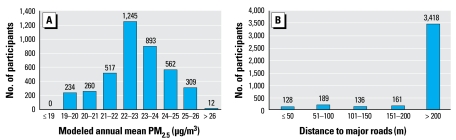
Distribution of modeled annual air pollution exposure (year 2002) and residential distance to major roads for 4,032 participants of the Heinz Nixdorf Recall Study.

Figure 2 illustrates the study region and locations of the participants’ residences. The study region includes areas with neighborhoods of multiunit residential apartment blocks transected by major highways, sub urban neighborhoods of mainly single- and double-unit houses, and rural areas in the south. Because of the strict definition of major road (freeways and federal highways), only about 15% of all participants lived within 50 m of a major road, which had mean daily vehicle counts of 10,000–130,000. Mean annual PM_2.5_ concentration modeled on a 5-km grid-cell length (year 2002) was 22.8 μg/m^3^ (range, 19.8–26.8 μg/m^3^) (Figure 3).

Markers of long-term exposure (annual mean PM_2.5_ and residential proximity to high traffic) were not correlated (Spearman correlation coefficient = 0.01). Daily ozone concentration and apparent temperature were moderately correlated with each other, but not with daily PM_10_ (Table 1).

Table 2 describes the statistics of the study population. Participants included in the main analysis (*n* = 4,032) did not differ systematically from those excluded (*n* = 782) regarding exposure and personal characteristics (data not shown).

In the complete study group, we observed an association of PM_2.5_ with the inflammatory mediators hs-CRP and fibrinogen. In the crude analysis and in the adjusted models 1 and 2, a 3.91 μg/m^3^ increase in PM_2.5_ was associated with an increase in hs-CRP of 16.7% [95% confidence interval (CI), 6.8 to 27.5%], 14.0% (95% CI, 0 to 30.0%), and 10% (95% CI, −2.6 to 24.3%), respectively. Using the same models, PM_2.5_ was associated with an increase in fibrinogen of 2.4% (95% CI, 0.6 to 4.2%), 3.69% (95% CI, 1.0 to 6.4%), and 2.7% (95% CI, 0.1 to 5.3%), respectively.

Table 3 displays the sex-stratified results. For men, effect estimates in the crude and the two adjusted models (models 1 and 2) were very similar, yielding a 24% increase in hs-CRP and a 4% increase in fibrinogen. Within the relatively small range of PM_2.5_ exposures in our study region, we did not find a positive exposure–response relationship, but all point estimates for the upper three quartiles of exposure were similarly elevated compared with the reference category of the lowest quartile.

In contrast to the findings in men, we found no consistent association of PM_2.5_ with inflammatory markers in women. We also observed no association between traffic exposure and systemic inflammatory markers in both men and women.

Including daily centrally measured air pollutants and apparent temperature attenuated the estimates for long-term exposure in men slightly (Table 4). Daily PM_10_ averaged over the 5 days preceding the blood draw was not associated with inflammatory markers. However, average ozone concentration and mean temperature during the 5 previous days were both associated with hs-CRP and fibrinogen.

For the association of long-term PM_2.5_ with inflammatory markers, we observed no distinct differences in effect size among subgroups defined by age, smoking status, BMI, diabetes, CHD, and current medication because CIs overlapped considerably (Table 5). However, in men, effect estimates were higher in older subjects, nonsmokers, and nonobese subjects for both hs-CRP and fibrinogen. In women, nonobese, diabetic subjects, and subjects with higher education showed higher estimates for PM_2.5_.

In the logistic regression model, long-term average PM_2.5_ was weakly associated with high levels (> 90th percentile) of hs-CRP and fibrinogen in men [crude odds ratio for cross-sectional difference of 3.91 μg/m^3^ PM_2.5_, 1.45 (95% CI, 0.99 to 2.12) for hs-CRP and 1.63 (95% CI, 1.11 to 2.41) for fibrinogen; adjusted odds ratio (model 2), 1.45 (95% CI, 0.80 to 2.64) for hs-CRP and 2.06 (95% CI, 1.12 to 3.78) for fibrinoge]. We found no associations with dichotomized inflammatory markers in women. Using different lags and averaging times for recent exposure to PM_10_, ozone, and temperature had no influence on the long-term PM_2.5_ estimate. Recent centrally measured PM_10_ was not associated with the inflammatory markers; however, we observed significant associations of recent ozone concentrations and apparent temperature with hs-CRP and fibrinogen.

In line with the hypothesis that effects of residential exposures should be stronger in subjects who presumably had spent more time at home before the baseline examination (smaller exposure estimation error), long-term PM_2.5_ was associated with inflammatory markers in men who had not been working within the past year, compared with subjects having worked any full-time job during this period [model 2, 48.0% (95% CI, 13.3 to 93.4%) vs. 12.2% (95% CI, −10.8 to 41.1%) increase in hs-CRP; 6.5% (95% CI, 0.7 to 12.7%) vs. 2.5% (95% CI, −2.2 to 7.4%) increase in fibrinogen]. In women, we observed no consistent pattern regarding time spent at home. This was also true for residential traffic exposure and inflammatory markers, both in men and in women.

Including covariates for current medication with lipid-lowering drugs, statins, NSAIDs, and a combined variable and covariates for comorbidities (CHD, diabetes mellitus, hypertension, chronic obstructive lung disease, and arthritis) did not substantially influence the long-term PM_2.5_ estimates. Excluding city and area of residence from the models decreased the estimates in men [model 1, 21.7% (95% CI, 7.6 to 37.7%) increase in hs-CRP and 2.64% (95% CI, 0.1to 5.2%) increase in fibrinogen; model 2, 14.0% (95% CI, 1.7 to 27.8%) increase in hs-CRP and 1.6% (95% CI, −0.8 to 4.0%) increase in fibrinogen], whereas it had an inconsistent effect in women. We observed no qualitative change in the exposure–response relation.

## Discussion

The key finding of our study is that long-term residential exposure to high levels of PM_2.5_ but not residential exposure to high traffic is weakly associated with systemic inflammatory markers in men, independent of acute changes in air pollution or temperature. Higher estimates in the subgroup of men with a presumably smaller exposure estimation error support our findings. We observed no consistent associations between levels of PM_2.5_ or residential traffic exposure and systemic inflammatory markers in women.

Our results indicate that, in men, chronic exposure to high levels of PM_2.5_ might induce a low-grade persistent systemic inflammatory state, which is independent of short-term changes in air pollutants and temperature. This is a novel finding, because in most studies only acute effects of short-term PM increases, which might trigger an acute cardiovascular event, have been investigated (Delfino et al. 2008; Dubowsky et al. 2006; Peters et al. 2001; Rückerl et al. 2007; Seaton et al. 1999; Yue et al. 2007). However, inflammation also plays an important role in the chronic process of atherogenesis (Libby 2002). Environmental inhalation exposures such as smoking or regular exposure to biomass smoke have been shown to induce a chronic low-grade inflammatory state (Baccarelli et al. 2007b; Ray et al. 2006). Few studies so far have examined effects of medium- or long-term PM exposures, showing weak associations with CRP (Diez Roux et al. 2006; Zeka et al. 2006) and white blood count (Chen and Schwartz 2008). Our findings provide a possible explanation for prior results indicating an association between long-term air pollution and subclinical markers of atherosclerosis (Diez Roux et al. 2008; Hoffmann et al. 2007; Künzli et al. 2005).

Remarkably, our results show a group difference between the lowest quartile of exposure and the upper three quartiles, without a clear exposure–response relationship within the upper three quartiles of exposure. Besides our lack of power to identify a possibly existing linear exposure–response relationship, given the widely overlapping CIs, this could also be explained by biologic processes following a nonlinear saturation kinetic, similar to mechanisms associated with ETS exposure (Pechacek and Bapp 2004).

Although we observed associations of long-term PM_2.5_ exposure with inflammatory markers, we did not find consistent associations of long-term exposure to high traffic with inflammatory markers. Urban background PM_2.5_, averaged in a grid cell of 25 km^2^, is dominated by larger and more stable fine PM, which has been shown to induce pulmonary inflammation with a secondary systemic inflammatory response (Frampton 2006). Residence close to a major road, however, leads to high exposure with traffic-related, combustion-derived UFPs (< 100 nm) (Zhu et al. 2002). UFPs can escape pulmonary defense mechanisms because of their small size and might be able to translocate into the systemic circulation (Elder and Oberdörster 2006), where they may directly access the endothelium and promote atherosclerosis (Yamawaki and Iwai 2006) without prior induction of a systemic inflammatory response. Furthermore, soluble components from PM such as metals or organic substances, originating to a high degree from traffic-related combustion, enter the systemic circulation, where they can act on the endothelium without inducing systemic inflammation (Wallenborn et al. 2007). Experimental evidence for the translocation of PM remains controversial, however, and exact knowledge about the cellular and molecular mechanisms of a possible PM-induced chronic pulmonary inflammation with a secondary low-grade systemic inflammatory state is still lacking. Other possible explanations for our inability to observe an effect of residential traffic exposure include exposure misclassification by using only distance to a major road instead of actual traffic density in the vicinity of the residence.

We also found no association with short-term PM_10_ exposure. Possible explanations include *a*) the low power of our cross-sectional study design to identify weak short-term effects, and *b*) misclassification of short-term exposures due to spatial variability of daily exposures within the study area.

Our findings suggest that the observed association is modified by several personal characteristics, the most obvious being the differences between men and women. Different sex-specific effects of air pollution with outcomes such as inflammatory markers and atherosclerosis have also been reported by others (Künzli et al. 2005; Steinvil et al. 2008). First, different exposure patterns and sources could explain some of the heterogeneity. Second, endogenous estrogen and post-menopausal hormone replacement therapy have been shown to alter plasma levels of a variety of cytokines and inflammatory markers, possibly contributing to the observed effect modification (Störk et al. 2008; Wong et al. 2008). In our own study population, sex differences in the association of hs-CRP and coronary artery disease have recently been documented (Erbel et al. 2008).

In our highly industrialized study area with still ongoing coal mining and steel production, men are more likely to be or have been employed in occupational settings with dusts and fumes, which might be independently associated with these two inflammatory markers. Therefore, confounding by occupational exposure could be a concern. However, when stratifying according to broad categories of SES, we found higher effect estimates in highly educated men, who are less likely to work in jobs with dust exposure. Associations were also more pronounced in participants who were older, nonobese, or nonsmokers. Diabetes, CHD, and cardiovascular or anti-inflammatory medication had no consistent influence on the effect estimates in men. In contrast to earlier findings in short-term exposure studies, we found no clear attenuation of effects by intake of statins or any other cardio vascular or antiinflammatory medication (Dubowsky et al. 2006).

Even though our effect estimates were generally inconsistent in women, we noted higher estimates in nonobese, highly educated, and diabetic subjects. The low precision of our estimates prohibit definite conclusions about factors that determine individual susceptibility; however, our findings warrant further investigations of sex-specific mechanisms and other individual characteristics that may play a role in the induction of chronic inflammation.

Because identifying a minimal sufficient adjustment set with the help of causal diagrams is strongly dependent on assumptions regarding the temporal and causal relations between variables, other multivariable models might be more adequate. We based our causal diagram on prior knowledge. However, other causal relations could be possible, resulting in different adjustment sets. Also, residual confounding in the adjustment for individual SES might be large. We therefore used two different approaches by adjusting for a nonminimal set of variables as well as excluding SES variables. Results for the long-term PM_2.5_ estimates were very similar, which strengthens our confidence in the applicability of the chosen DAG for the investigation of long-term urban background PM exposures.

In the analyses of residential proximity to major roads, estimates using the two different adjustment sets differed substantially, with estimates for the SES adjustment set (model 1) being closer to the crude estimate and the estimates from model 2, comprising lifestyle related factors and age instead of SES variables, were usually closer to the null. This could point to an inadequately specified causal model for the assessment of effects due to very small-scale spatial variability in traffic exposure.

The major limitation of our study is the relatively small exposure contrast for PM_2.5_ in our study population, caused by the degree of spatial resolution our current air quality model provides. In this analysis, we used a grid cell length of 5 km, resulting in cell sizes of 25 km^2^, which probably leads to substantial exposure estimation error in this metropolitan area. Future analyses with a higher spatial resolution of the air quality model will enable us to examine the size and effect of this potential source of exposure estimation error more closely. Moreover, our long-term exposure assessment is inhibited by a missing temporal resolution. We used the 2002 annual PM_2.5_ average, regardless of the actual dates of the subjects’ baseline examinations. This exposure measure can lead to an error in the exposure assessment if yearly or monthly trends in exposure are different across the study region.

Major strengths of our study include the availability of measures of long-term as well as short-term exposure to ambient PM air pollution and the possibility to adjust for short-term changes in temperature and ozone exposure. Moreover, the geographically small and coherent study region probably provided a more similar mixture of PM constituents throughout the study region than in studies pooling data from regions further apart and where PM effects might be confounded by different compositions of the PM mixture. In addition, the large, population-based sample of well-examined participants allowed extensive control of possible confounding covariates.

In conclusion, we have shown a weak association between long-term PM_2.5_ exposure and systemic inflammatory markers in men, which is independent of short-term changes in air pollution or temperature. This possibly indicates the induction or sustainment of a low-grade systemic inflammatory state by chronic PM_2.5_ exposure. Considering the role of these markers as potential mediators in the pathogenesis of atherosclerotic disease and the known relation between long-term PM air pollution and cardiovascular events, this result provides a link between long-term PM exposure and the hypothesized development and progression of atherosclerosis.

## Figures and Tables

**Table 1:** Description of centrally measured daily air pollutant concentrations and mean temperature for the period of the baseline analysis of the Heinz Nixdorf Recall Study (December 2000–July 2003).

					Correlation coefficient	
	No.	Mean ± SD	Minimum	Maximum	Mean temperature	Ozone
Daily PM_10_ (μg/m^3^)	803	37.7 ± 19.7	5.0	147.0	0.04	−0.12
Daily ozone (μg/m^3^)	954	34.1 ± 21.5	2.0	111.0	0.60	—
Daily mean temperature (°C)	971	0.3 ± 6.8	−8.7	26.8	—	—

**Table 2:** Descriptive statistics of the study population (*n* = 4,032).

	Total^a^ (*n* = 4,032)	Men (*n* = 2,028)	Women (*n* = 2,004)
Age [years (mean ± SD)]	60.0 ± 7.8	60.1 ± 7.7	59.9 ± 7.8
Proximity to major road [*n* (%)]			
> 200 m	3,481 (84.8)	1,716 (84.6)	1,702 (84.9)
> 100–200 m	297 (7.4)	152 (7.5)	145 (7.2)
> 50–100 m	189 (4.7)	95 (4.7)	94 (4.7)
≤ 50 m	128 (3.2)	65 (3.2)	63 (3.1)
City [*n* (%)]			
Mülheim	1,501 (37.2)	759 (37.4)	742 (37.0)
Essen	1,338 (33.2)	655 (32.3)	683 (34.1)
Bochum	1,193 (29.6)	614 (30.3)	579 (28.9)
Area of residence [*n* (%)]			
North	562 (13.9)	275 (13.6)	287 (14.3)
Central	2,272 (56.4)	1,172 (57.8)	110 (54.9)
South	1,198 (29.7)	581 (28.7)	617 (30.8)
Educational level [*n* (%)]			
≤ 10 years	415 (10.3)	96 (4.7)	319 (15.9)
11–13 years	2,243 (55.6)	941 (46.4)	1,302 (65.0)
14–17 years	928 (23.0)	700 (34.5)	228 (11.4)
≥ 18 years	446 (11.1)	291 (14.4)	155 (7.7)
Economic activity [*n* (%)]			
Employed	1,659 (41.2)	982 (48.4)	677 (33.8)
Inactive, homemaker	541 (13.4)	8 (0.4)	533 (26.6)
Retired	1,585 (39.3)	929 (45.8)	656 (32.7)
Unemployed	247 (6.1)	109 (5.4)	138 (6.9)
Smoking status [*n* (%)]			
Daily smoker	825 (20.5)	432 (21.3)	393 (19.6)
Occasional smoker	104 (2.6)	67 (3.3)	37 (1.9)
Ex-smoker, ≤ 1 year	73 (1.8)	46 (2.3)	27 (1.4)
Ex-smoker, > 1 year	1,359 (33.7)	915 (45.1)	444 (22.2)
Never-smoker	1,671 (41.4)	568 (28.0)	1,103 (55.0)
ETS [*n* (%)]	1,308 (32.4)	697 (34.4)	611 (30.5)
BMI [kg/m^2^ (mean ± SD)]	27.9 ± 4.6	28.2 ± 3.9	27.7 ± 5.2
Waist circumference [cm (mean ± SD)]	94.4 ± 13.1	100.2 ± 10.7	88.5 ± 12.8
Weekly physical activity [*n* (%)]	2,057 (51.0)	1,044 (51.5)	1,013 (50.6)
Regular alcohol intake [*n* (%)]	819 (20.3)	605 (29.8)	214 (10.7)
Diabetes mellitus [*n* (%)]	555 (13.8)	363 (17.9)	192 (9.6)
CHD [*n* (%)]	270 (6.7)	216 (10.7)	54 (2.7)
Any antiinflammatory drugs [*n* (%)]^b^	1,629 (40.4)	852 (42.0)	777 (38.8)
Total cholesterol [mg/dL (mean ± SD)]	229 ± 39	224 ± 38	233 ± 40
Hs-CRP [mg/L, median (interquartile range)]	1.50 (2.50)	1.50 (2.40)	1.50 (2.50)
Fibrinogen [mg/dL, median (interquartile range)]	324 (97)	317 (95)	332 (98)

a Excludes participants with incomplete data on outcome or explanatory variables (782).

b Includes statins, NSAIDs, angiotensin-converting enzyme inhibitors, and beta-blockers.

**Table 3:** Associations of long-term PM_2.5_ exposure and residential traffic exposure with markers of inflammation [% change (95% CI)].

	hs-CRP			Fibrinogen		
	Crude model	Model 1^a^	Model 2^b^	Crude model	Model 1^a^	Model 2^b^
Men (*n* = 2,028)						
PM_2.5_ (per 3.91 μg/m^3^)	27.2 (12.2 to 44.1)	29.4 (7.3 to 56.1)	23.9 (4.1 to 47.4)	3.5 (0.9 to 6.1)	4.8 (0.9 to 8.8)	3.9 (0.3 to 7.7)
PM_2.5_ by quartiles^c^						
> Q1–Q2	24.6 (6.8 to 45.3)	21.8 (1.7 to 45.9)	19.4 (1.0 to 41.1)	4.0 (0.8 to 7.3)	2.7 (−0.9 to 6.5)	2.0 (−1.5 to 5.6)
> Q2–Q3	13.6 (0.1 to 28.9)	17.2 (−2.4 to 40.8)	17.1 (−1.3 to 38.8)	1.4 (−1.1 to 4.1)	2.4 (−1.3 to 6.2)	2.8 (−0.8 to 6.4)
> Q3	38.2 (19.8 to 59.4)	22.7 (0.7 to 49.3)	18.6 (−1.1 to 42.3)	5.4 (2.4 to 8.5)	4.1 (0.1 to 8.3)	3.7 (−0.1 to 7.7)
Proximity to major road^d^						
> 100–200 m	21.1 (0.5 to 45.8)	16.5 (−3.1 to 40.0)	11.9 (−5.7 to 32.7)	1.6 (−2.1 to 5.5)	0.9 (−2.8 to 4.7)	0.5 (−3.0 to 4.1)
> 50–100 m	5.9 (−16.0 to 33.5)	1.4 (−19.3 to 27.5)	−1.7 (−20.5 to 21.4)	0.6 (−4.0 to 5.4)	0.0 (−4.5 to 4.7)	−0.6 (−4.8 to 3.9)
≤ 50 m	20.2 (−9.0 to 58.6)	18.4 (−10.0 to 55.7)	2.9 (−20.0 to 32.7)	3.2 (−2.4 to 9.1)	3.2 (−2.3 to 9.0)	1.1 (−4.1 to 6.6)

Women (*n* = 2,004)						
PM_2.5_ (per 3.91 μg/m^3^)	6.6 (−5.9 to 20.8)	0.4 (−16.5 to 20.7)	−1.0 (−16.5 to 17.3)	1.2 (−1.3 to 3.8)	2.5 (−1.3 to 6.3)	1.5 (−1.9 to 5.1)
PM_2.5_ by quartiles^c^						
> Q1–Q2	7.2 (−7.6 to 24.3)	7.2 (−11.0 to 29.1)	3.2 (−13.1 to 22.6)	2.1 (−0.9 to 5.1)	1.3 (−2.4 to 5.2)	0.7 (−2.8 to 4.3)
> Q2–Q3	2.7 (−8.3 to 16.3)	6.9 (−10.5 to 27.8)	8.6 (−7.9 to 27.9)	0.6 (−1.9 to 3.1)	2.4 (−1.2 to 6.1)	2.0 (−1.4 to 5.5)
> Q3	12.7 (−2.0 to 29.5)	2.0 (−15.1 to 22.6)	2.0 (−13.9 to 20.9)	2.4 (−0.5 to 5.2)	2.6 (−1.1 to 6.5)	1.7 (−1.8 to 5.3)
Proximity to major road^d^						
> 100–200 m	8.6 (−9.7 to 30.7)	5.4 (−12.4 to 26.9)	7.2 (−9.6 to 27.0)	2.1 (–1.6 to 6.0)	1.7 (−2.0 to 5.5)	1.7 (−1.8 to 5.3)
> 50–100 m	−2.6 (−22.4 to 22.2)	−6.0 (−25.0 to 17.7)	−14.4 (−30.5 to 5.4)	−4.3 (−8.5 to 0.1)	−4.7 (−8.9 to −0.3)	−6.1 (−10.0 to –2.0)
≤ 50 m	5.2 (−20.0 to 38.4)	−1.0 (−24.6 to 30.0)	−15.3 (−34.1 to 8.9)	2.2 (−3.3 to 7.9)	1.3 (−4.1 to 6.9)	−0.6 (−5.6 to 4.6)

a Model 1 represents the minimal sufficient adjustment set, including city and area of residence and SES variables (education and economic activity).

b Model 2 represents the nonminimal adjustment set without SES variables. Covariates are city and area of residence, age, smoking variables, ETS, physical activity, alcohol intake, BMI, waist circumference, LDL, and HDL.

c Lowest quartile used as reference. Q1, 21.54 μg/m^3^; Q2, 22.59 μg/m^3^; Q3, 23.75 μg/m^3^.

d > 200 m used as reference.

**Table 4:** Estimates for the association of modeled annual PM_2.5_, centrally measured daily air pollutants (PM_10_, ozone), and mean daily air temperature with inflammatory markers [% change (95% CI)].^a^

	hs-CRP		Fibrinogen	
	Model 2	Model 2 plus daily exposures	Model 2	Model 2 plus daily exposures
Men (1,752)^b^				
Annual PM_2.5_ (per 3.91 μg/m^3^)	23.7 (2.4 to 49.5)	19.4 (−1.1 to 44.2)	3.2 (−0.7 to 7.3)	2.2 (−1.7 to 6.2)
5-day mean PM_10_ (per 31.85 μg/m^3^)	—	1.9 (−9.1 to 14.0)	—	−0.8 (−3.0 to 1.6)
2-day mean ozone (per 55 μg/m^3^)	—	20.8 (1.7 to 43.6)	—	2.2 (−1.3 to 5.9)
5-day mean temperature (per 16.98 °C)	—	−17.6 (−29.4 to –3.8)	—	−6.3 (−9.2 to −3.3)

Women (1,716)^b^				
Annual PM_2.5_ (per 3.91 μg/m^3^)	−0.7 (−17.4 to 19.4)	−2.0 (−18.5 to 17.9)	2.0 (–1.8 to 5.8)	2.2 (−1.5 to 6.1)
5-day mean PM_10_ (per 31.85 μg/m^3^)	—	−7.1 (−17.1 to 4.0)	—	−1.4 (−3.6 to 0.1)
2-day mean ozone (per 55 μg/m^3^)	—	28.1 (7.7 to 52.4)	—	2.4 (−1.1 to 6.1)
5-day mean temperature (per 16.98°C)	—	−14.8 (−27.0 to −0.5)	—	−5.1 (−8.0 to −2.1)

a Based on a restricted sample of 1,752 men and 1,716 women because of missing data on daily exposure variables.

b Estimates adjusted for city and area of residence, age, smoking variables, ETS, physical activity, alcohol intake, BMI, waist circumference, LDL, and HDL (model 2 from Table 3) and long-term time trend.

**Table 5:** Adjusted estimates (model 2) of subgroup analyses for PM_2.5_ exposure and inflammatory markers CRP and fibrinogen [% change (95% CI)].

	Men (*n* = 2,028)		Women (*n* = 2,004)	
	hs-CRP	Fibrinogen	hs-CRP	Fibrinogen
Age < 60 years	17.7 (−8.4 to 51.2)	3.6 (−1.5 to 8.9)	10.4 (−13.6 to 41.0)	2.1 (−2.9 to 7.3)
Age ≥ 60 years	32.6 (3.8 to 69.4)	5.0 (−0.3 to 10.6)	−10.2 (−29.2 to 13.8)	1.1 (−3.7 to 6.2)
Low SES	15.5 (−9.7 to 47.8)	5.0 (−0.3 to 10.6)	−7.6 (−23.5 to −11.6)	0.0 (−3.9 to 3.9)
High SES	34.0 (4.2 to 72.3)	3.3 (−1.9 to 8.7)	23.9 (−18.0 to 85.1)	9.0 (1.0 to 17.7)
Nonsmoker	32.8 (8.7 to 62.1)	4.4 (0.3 to 8.7)	−1.1 (−18.0 to 19.4)	2.4 (−1.4 to 6.5)
Smoker	0.1 (−30.1 to 43.4)	1.9 (−5.7 to 10.2)	−0.2 (−32.5 to 47.7)	−2.1 (−9.6 to 6.0)
BMI < 30 kg/m^2^	28.7 (4.5 to 58.5)	4.7 (0.4 to 9.1)	7.0 (−13.2 to 32.0)	3.1 (−1.1 to 7.5)
BMI ≥ 30 kg/m^2^	12.0 (−18.7 to 54.3)	1.8 (−5.4 to 9.5)	−15.9 (−37.0 to 12.2)	−1.6 (−7.7 to 4.9)
No diabetes	23.6 (2.0 to 49.7)	5.0 (1.0 to 9.2)	−9.4 (−24.2 to 8.4)	0.5 (−3.1 to 4.3)
Diabetes	21.6 (−20.2 to 85.2)	−0.7 (−9.2 to 8.5)	82.6 (1.1 to 230.3)	8.6 (−3.6 to 22.4)
No CHD	24.1 (3.7 to 48.6)	4.2 (0.5 to 8.2)	1.6 (−14.4 to 20.7)	1.6 (−1.9 to 5.2)
CHD	36.9 (−27.5 to 158.5)	3.1 (−10.0 to 18.0)	−52.4 (−86.2 to 63.3)	−2.6 (−20.7 to 19.5)
No medication	25.0 (0.0 to 56.4)	5.6 (1.0 to 10.5)	3.9 (–16.4 to 29.2)	1.3 (−3.1 to 5.8)
Any medication^a^	28.3 (−3.0 to 69.8)	2.6 (−3.3 to 8.9)	−10.2 (−31.7 to 18.0)	1.8 (−3.8 to 7.7)

a Includes statins, NSAIDs, angiotensin-converting enzyme inhibitors, and beta-blockers.
